# Innovative solutions to novel drug development in mental health

**DOI:** 10.1016/j.neubiorev.2013.03.022

**Published:** 2013-12

**Authors:** T.R. Insel, V. Voon, J.S. Nye, V.J. Brown, B.M. Altevogt, E.T. Bullmore, G.M. Goodwin, R.J. Howard, D.J. Kupfer, G. Malloch, H.M. Marston, D.J. Nutt, T.W. Robbins, S.M. Stahl, M.D. Tricklebank, J.H. Williams, B.J. Sahakian

**Affiliations:** a National Institute of Mental Health, National Institutes of Health, Bethesda, MD, USA; b Department of Psychiatry, University of Cambridge, Cambridge, UK; c MRC/Wellcome Trust Behavioral and Clinical Neuroscience Institute, University of Cambridge, Cambridge, UK; d Neuroscience, Janssen Pharmaceutical of Johnson and Johnson, Titusville, NJ, USA; e School of Psychology and Neuroscience, St Andrews University, St Andrews, UK; f Institute of Medicine, Forum on Neuroscience and Nervous System Disorders, Washington, DC, USA; g Glaxo Smith Kline, Clinical Unit, Addenbrookes Hospital, Cambridge, UK; h Department of Psychiatry, University of Oxford, Oxford, UK; i Royal College of Psychiatrists and Institute of Psychiatry, Kings College London, London, UK; j University of Pittsburgh School of Medicine, Department of Psychiatry, Pittsburg, PA, USA; k Medical Research Council, UK; l TPP Global Development, Edinburgh, UK; m Centre for Pharmacology and Therapeutics, Division of Experimental Medicine, Imperial College London, London, UK; n Department of Psychology, University of Cambridge, Cambridge, UK; o Department of Psychiatry, University of California, San Diego, USA; p Eli Lilly and Co Ltd, Windlesham, UK; q Wellcome Trust, UK

**Keywords:** Translation, Back-translation, Pharmacological tool box, Cognitive and psychosocial treatments, Novel drug development, Biomarkers, Neurobiological mechanisms, Neuropsychiatric disorders

## Abstract

There are many new advances in neuroscience and mental health which should lead to a greater understanding of the neurobiological dysfunction in neuropsychiatric disorders and new developments for early, effective treatments. To do this, a biomarker approach combining genetic, neuroimaging, cognitive and other biological measures is needed. The aim of this article is to highlight novel approaches for pharmacological and non-pharmacological treatment development. This article suggests approaches that can be taken in the future including novel mechanisms with preliminary clinical validation to provide a toolbox for mechanistic studies and also examples of translation and back-translation. The review also emphasizes the need for clinician-scientists to be trained in a novel way in order to equip them with the conceptual and experimental techniques required, and emphasizes the need for private-public partnership and pre-competitive knowledge exchange. This should lead the way for important new holistic treatment developments to improve cognition, functional outcome and well-being of people with neuropsychiatric disorders.

## Highlights

• New approaches to identifying and validating potential drug targets are essential.
• A novel drug toolbox and innovative private-public partnerships are required.
• Translational neuroscience research will enhance understanding of mental health.
• The training of a new cadre of clinician scientists is a necessary foundation.
• Neuropsychiatric disorders require holistic, early, effective treatment.

The last decade has witnessed exciting and important advances in the neuroscience of mental health including the mapping of neural circuitry and neurochemical mechanisms, identification of multiple genetic loci and the application of novel technologies to both the pathophysiology and treatment of mental disorders. Despite these advances, major unmet needs remain. Mental illness remains the leading cause of morbidity and mortality (Bloom et al., 2011; Collins et al., 2011; Insel, 2009). Psychiatric conditions account for five of the top ten causes of disability and premature death and mental health conditions are the leading cause of Disability Adjusted Life Years accounting globally for 37% of healthy life years lost from Non-Communicable Diseases. The global cost for disorders of mental health in 2010 was $2.5 trillion and projected to markedly increase to $6.5 trillion in 2030, making mental illness the most costly form of chronic disease worldwide (Bloom et al., 2011). Furthermore, a considerable proportion of people with mental health problems remain untreated. For example, in the USA 67% and in Europe 74% of people with mental illness are untreated. (Thornicroft, 2007) Yet, in spite of these urgent unmet needs, mental health is experiencing a crisis in the development of new treatments, especially drug treatments. In the last 40 years, very few therapeutics with novel mechanisms have progressed to phase III clinical trials or regulatory approval. Major pharmaceutical companies are even shifting drug discovery efforts away from psychiatric toward non-psychiatric disorders with identified biological targets (Cressey, 2010; Miller, 2010). This issue of private sector drug development is one major symptom reflecting deeper underlying infrastructural issues in mental health research. The Royal Society recently convened an International Scientific Seminar to find innovative solutions for novel drug development. The meeting concluded that to address these issues, we require a paradigm shift in how we: diagnose and categorize psychiatric disorders, view and approach mental health research, encourage collaborative partnership models between academia and drug companies, train the next generation of clinicians, maintain the pre-clinical knowledge base and influence the public perception of mental illness. The following seeks to address these fundamental problems and to propose a way forward for the next two decades.

## 1 Many psychiatric disorders are neurodevelopmental in origin

**Fig. 1:**
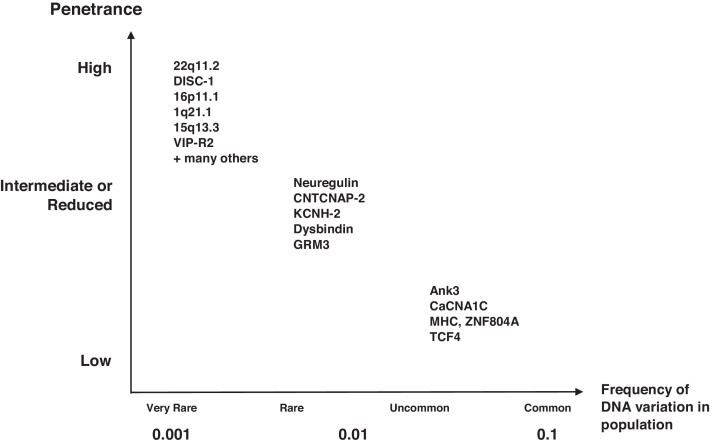
Complex genetics of mental disorders: prevalence and risk. This figure outlines genetic risk factors and rare genetic aetiologies that may be appropriate for population based screening and translation into therapeutic approaches. Three categories are identified: highly penetrant or high risk but rarely identified; more commonly identified through genomic sequencing; and higher prevalence but low contribution to risk. The degrees of penetrance are defined as follows: highly penetrant (the trait or symptom will almost always be expressed in those carrying the allele); incomplete or reduced (some individuals fail to express the trait despite carrying the allele); low penetrance (an allele will only sometimes produce the symptom).

Psychiatric disorders are brain disorders of complex and variable genetic risk interacting with neural circuitry and experience. Mental disorders disproportionately affect the young with 75% of illnesses having onset before the age of 24 (Kessler et al., 2005). The identification of multiple genetic loci for complex disorders exploded in the decade following the sequencing of the first human genome, with 2850 disease genes identified for Mendelian-based disorders and 1100 loci identified for 165 common multigenic diseases as of February, 2011 (Lander, 2011). Accordingly, in the most heritable neuropsychiatric disorders (autism, schizophrenia, bipolar disorder) at least a dozen risk alleles have been reported from genome wide association studies, including many common variants replicated recently in a global effort with over 100,000 subjects across 65 research institutions (Fig. 1) (Ripke et al., 2011; Sklar et al., 2011). Supporting the concept of mental disorders as neurodevelopmental, several of these apparent risk loci are key factors in neurodevelopmental pathways. In addition to the genomic evidence, longitudinal imaging studies have demonstrated altered patterns of development in patients with mental disorders. For instance, children with attention deficit hyperactivity disorder show a profound and consistent delay in cortical maturation (Shaw et al., 2007). These kinds of findings have led to a reconceptualization of mental disorders as brain disorders resulting from the aberrant development of specific circuits. This reconceptualization is exemplified in a new model of major depression which proposes different nodes for the underlying circuits, with alterations in neural pathways for emotion, cognition, interoception, and self-awareness (Drevets et al., 1997; Ressler and Mayberg, 2007). These pathways not only suggest a new stratification for depression, they may provide differential targets for medications, cognitive behavioral therapy, and deep brain stimulation. (Drevets and Furey, 2010; Holtzheimer and Mayberg, 2011; Zarate et al., 2006) Initiatives such as The Human Connectome Project (www.humanconnectomeproject.org) and the 1000 Connectomes Project (www.fcon_1000.projects.nitrc.org) which are mapping the variation in whole brain structural and functional network organization through large scale data sharing schemes should yield a consensus wiring diagram of the human brain and a range of individual variation, analogous to the maps of common and uncommon variation in the human genome.

### 1.1 Challenges in drug development

Despite these major advances in knowledge, progress in the search for novel therapeutic compounds has been difficult. Several inter-related factors account for this failure. Thus far, genetics has not uncovered druggable targets for mental disorders. The many variants identified have conspicuously not revealed targets related to monoamines, suggesting that genetics may take us beyond the cluster of current drugs, but we will need to bridge the gap between genetic findings and targets. An additional challenge is that the disease state remains based on phenomenological rather than biological categories, with limited understanding of pathophysiology. Additionally, there is a need for breakthrough clinical insights. The development of an antihistaminergic compound into chlorpromazine as an antipsychotic and imipramine as an antidepressant in the 1950s was a major novel development that revolutionized treatment in psychiatry. While we are likely to dismiss these discoveries in mental health as the result of serendipity and careful observation rather than anchored in established rational mechanistic processes, there is no reason to assume that careful clinical insights will not yield important therapeutic innovations in the future. Nevertheless, without a clear understanding of the biological basis of a disorder it will certainly be more difficult to find a rational approach to novel treatments. An example of hypothesis-driven drug development was that of the cholinesterase inhibitors in treating cognitive symptoms in dementias. This development was based on a pathological hypothesis derived from neuropathology and clinical analysis with utilization of existing pharmacological tools to validate the target. The result is a class of compounds particularly useful in improving attention and concentration, in patients with mild to moderate Alzheimer's disease (Eagger et al., 1991) but clearly more effective treatments are required particularly for episodic memory symptoms and neuroprotection.

A constellation of factors including the absence of molecular targets for drug discovery, the increasing cost and average duration of treatment discovery, and increasing placebo response rate and failure rates in clinical trials has led us to this crisis in drug development. (Nutt and Goodwin, 2011) Due to these challenges, a wealth of compounds interacting with promising targets have been developed by drug companies but lack convincing evidence of efficacy and are generally not available for widespread research by academic investigators. At the same time that we are facing a profound unmet need for new treatments and unprecedented scientific progress, research and development in industry is moving elsewhere, risking a lost generation for new treatment development.

### 1.2 Novel approaches for drug development

How can we address these fundamental issues? Our goals and perspective of mental health must change. Understanding molecular mechanisms will allow the identification of novel therapeutic targets including that of circuitry, genomics and epigenomics. Genetic findings in psychiatry, especially highly penetrant genetic lesions, need to advance to define new molecular targets. Unlike other fields where tissue biopsies or tumor removal has routinely been used to study pathophysiology and create cellular models of disease for testing new therapeutics, in psychiatric disorders brain tissue is rarely available during life. Functional and structural imaging, electrophysiology, and blood and cerebrospinal fluid-based measurements might yield glimpses into underlying pathological processes, especially when applied longitudinally during the years of risk and prodromal stages. Recently, the advent of skin-derived stem cells, also known as induced pluripotent cells (iPSc), that can be converted into neurons and glia in vitro promises to unveil pathogenetic mechanisms. (Tobe et al., 2011) Not only will iPScs create a “disease in a dish”, these individualized cultures can serve as substrates for high throughput screening and testing novel therapeutics. Indeed, early results using iPSc's in genetically determined neurological disorders such as Rett's syndrome, Parkinson's disease (LRRK2-linked) and Spinal muscular atrophy has confirmed that such cultures can mimic known cellular defects faithfully, raising hopes for neurodevelopmental conditions like schizophrenia with more complex etiologies. (Brennand et al., 2011)

How can we progress from genetic signals to molecular targets? In other fields, genetics is beginning to yield new targets. For example, *BCL11A* is a new target for drug development in sickle cell anemia. In people with a common variant that decreases expression of *BCL 11A*, a transcriptional repressor, fetal hemoglobin production is not repressed during development allowing enhanced oxygen carrying capacity even in those with the sickle cell mutation of adult hemoglobin. Thus, the blockade of this repressor of fetal hemoglobin represents a potentially novel therapeutic target. (Sankaran et al., 2008) Identifying such molecular mechanisms in mental disorders is crucial to the development of new biological targets. But if mental disorders are like other medical disorders, we may need to look for variants that are protective, where loss of function reduces risk. Furthermore, in addition to the complex technical issues of molecular targeting, it will need to address safety issues.

We need new approaches to identify risk factors or prodromal signs early enough in the course of illness to intervene before a disorder becomes a disability. Neurocognitive assessments and neuroimaging have already been applied to the identification of the prodrome of schizophrenia and early phases of autism. Genetics could also inform risk, even before the prodrome. While we are not ready for genetic screening for one of the many common risk alleles in neonates or young children, the detection of rare variants that are highly penetrant may already be clinically useful in some settings. These approaches suggest a transformation of diagnosis that considers risk states as well as symptomatic phases of the illness, as adopted in cardiology and oncology. Clearly, we need predictive biomarkers to identify an individual's vulnerability and resilience and provide markers for personalized and targeted therapies. And importantly, treatments for these high risk states are likely to be psychosocial (family support, cognitive training) rather than biomedical. There will of course be important ethical issues associated with potential stigma, the risk of false positives and other consequences of genetic screening.

In addition to considering the opportunities for treatment of prodromal and possibly high risk states, a new approach to symptomatic disorders could yield innovative ways of approaching treatment development (Sahakian et al., 2010). The Diagnostic and Statistical Manual for Mental Disorders is currently for clinical and research use. The Research Domain Criteria (RDoC) is a new effort that focuses on dimensions that cut across disease categories focusing on core domains of functioning that map on to clinical neural circuits and genetics as well as preclinical studies of brain and behavior (Insel et al., 2010; Sanislow et al., 2010). The RDoC is currently used as a research tool which may identify new clinical targets, such as anhedonia or social deficits, that can become new therapeutic endpoints. In the future, with further understanding of the underlying neurobiology and pathophysiologically relevant processes in animals and humans, the RDoC approach may well be for clinical use.

Beyond identifying new molecular targets and new clinical targets, we need to expect more of the next generation of therapeutics. Our treatment targets have been focused too much on symptom relief and too little on recovery; too much on treating the late stages of illness and too little on pre-emptive therapies during the prodromal stage (Beddington et al., 2008; Collins et al., 2011; Sahakian et al., 2010). For recovery, treatment needs to consider compliance, which remains a pervasive problem with current medications. To decrease morbidity and mortality, treatments need to be embedded in comprehensive medical care with supports for social and occupational function. And rather than a single magic bullet, our therapeutic targets should shift toward combination and integrative therapies which can combine pharmacological, psychosocial therapies and neurotechnologies. The use of d-cycloserine and CBT for phobias exemplifies such an effective combination therapy (Ressler et al., 2004). Other novel techniques and applications of new technology include the use of training through video games which may enhance cognitive performance in the prodromal stage of schizophrenia or increase eye contact in children with autism (Sahakian, 2011; Sahakian et al., 2010). Similarly, the rapid response of major depression to ketamine along with the identification of biomarkers of response emphasizes the promise of novel therapeutics (Diazgranados et al., 2010).

In addition to changing what we do, we should consider changes in how we develop the next generation of therapeutics. Innovative, collaborative partnerships are already being forged between academia and industry, recognizing that the old models are not going to be sufficient for future success. The development of a novel opioid receptor inverse agonist for overeating provides an effective example of a novel risk/reward sharing partnership between academia and a private sector partner (Rabiner et al., 2011).

A ‘fast-fail’ approach should be emphasized but must be balanced with comprehensive and thorough profiling across disease dimensions with adequate statistical power to dispel doubt. Greater communication and exchange of knowledge between academic and industry researchers will allow a sharing of existing and even new compounds. Joint development of validated methodology and infrastructure is likely to lead more quickly to new drug registrations and is more suited to current business models. There is an unprecedented increase in willingness of companies to engage in data and compound sharing, as exemplified by the European Union/European Federation of Pharmaceutical Industries and Associations Innovative Medicines Initiative (www.imi.europa.eu) and the European College of Neuropsychopharmacology medicine chest. (www.ecnp.eu/projects-initiatives/ECNP-medicines-chest.aspx) Arch2POCM is a new effort engaging several industry and academic scientists in a new paradigm for neuroscience drug development, redefining precompetitive space to enhance sharing, including translational models, data and samples.

Paradoxically, the reduction in Pharma investment in central nervous system disorders could be a boon for academic scientists. Compounds in industry that are not currently actively investigated could be made available for further non-clinical investigation, and if safe and fully qualified for clinical studies, should be made available for further mechanistic studies. There may now be fewer impediments to ‘repurposing’ and ‘rescue’. These approaches leverage existing investment and knowledge leading potentially to a lower risk and faster return. From a patient's perspective, focusing on mechanisms underlying side effect burden in chronic treatments in order to retain efficacy but improve acceptability and wellbeing would be a major advance leading to greater compliance and therefore reduced costs.

**Fig. 2:**
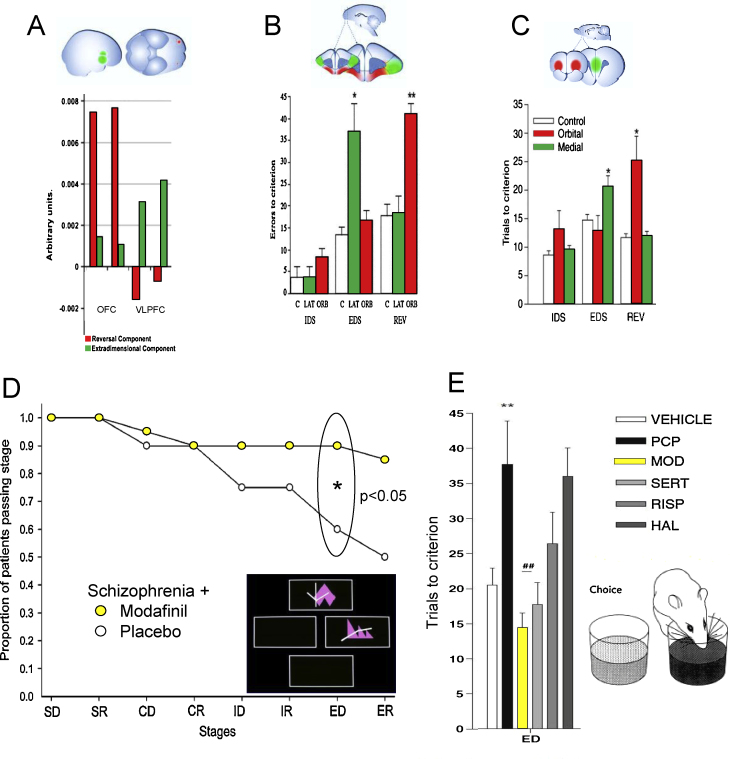
Translation and back-translation: cognitive flexibility. Intra-dimensional and extra-dimensional (ID/ED) set shifting tasks assess reversal learning (response to shift in outcome contingency) and attentional set-shifting (response to shift in focus of attention within the same dimension (IDS) or a different dimension (EDS)). (A-C) Translational studies: Dissociation of reversal learning and extra-dimensional set shifting as a function of prefrontal cortical sectors has been demonstrated in 4 species: (A) human; (B) marmoset; (C) rat and (not illustrated) mouse. Graphs for the marmoset and rat show the number of trials to criterion for ID, ED and Reversal learning in control (white), orbitofrontal cortex (red) and rodent medial prefrontal cortex and marmoset lateral prefrontal cortex (green). Graph for human shows fMRI units for Reversal and EDS in orbitofrontal cortex (red) and ventrolateral prefrontal cortex (green). These images are adapted from (Brown and Bowman, 2002; Dias et al., 1997; Hampshire and Owen, 2006). (D) The graph shows the improvement in EDS (*p < 0.05) when patients with schizophrenia are given modafanil (yellow) versus placebo. This graph is adapted from Turner et al. (2004). (E) Back-translation: In rats, PCP (black) worsens EDS compared to vehicle (white). This deficit is ameliorated by modafanil (yellow). The effects of sertindole, risperidone and haloperidol are also shown. This graph is adapted from (Goetghebeur and Dias, 2009).

Experimental medicine approaches are not new, however and repeating the best efforts of company-directed clinical trials via academic or academic-industrial consortia is unlikely to be game-changing without including a new strategy. Pharmacokinetic, toxicological and biomarker optimization for novel compounds and targets is the strength of the pharmaceutical industry. Predicting the potential beneficial effects of compounds from preclinical profiling methods that have translational predictive validity is neither a current strength of the industry nor of academia and is arguably the key reason why the late stage drug development pipeline is dry. We need to consider animal disease models and functional assays in as much a new way as we do clinical investigation following the removal of the constraints of classical psychiatric disease diagnosis: animal models of schizophrenia will never recapitulate all the disease symptoms but the biological dimensions of arousal, reward, motivation and cognition and the circuitries that underlie them can be accessed in animals now more easily than ever before and can be used to identify molecular control points – as well as chemical ligands that can modulate them. A paradigmatic example of successful translation and back-translation focusing on a core cognitive dimension is described in Fig. 2. This approach to addressing effective translation underlies the Cognitive Neuroscience Treatment Research to Improve Cognition in Schizophrenia (CNTRICS) initiative (Carter et al., 2008). The job of experimental medicine then becomes one of focussing the clinical investigation of the chemical ligands, or hopefully medicines, across these same dimensions in a systematic way in both volunteers and in patients. Experimental medicine thus needs to consider ways of quantitatively phenotyping patients and those at high-risk along these same dimensions with both clinical and preclinical scientists harnessing novel biomarker technologies (functional and structural imaging, connectivity, electrophysiology, cognitive paradigms, serological and immunological markers and (epi)genomics) in order to maximize convergent validity.

Funding agencies such as the Wellcome Trust and Medical Research Council have recently been targeting the deficiencies in drug development by providing funding for target validation, developing candidate therapeutic agents and Proof of Concept trials. Awards are targeted at collaborations between academia and industry. Recent funding schemes focus on promoting experimental medicine and increasing research capacity and training through the support of multi-disciplinary research groups, enhancing research training and adding incentive for research careers in mental health research. Support for the development of respositories of large scale datasets and data sharing encourages the development of population health data. In the United States, the National Institutes of Health (NIH) has proposed the formation of a new institute, the National Center for Advancing Translational Science (NCATS), specifically to support research on the discipline of translation, identifying and overcoming roadblocks to the development of new treatments.

To bring this vision forward, the training of a new cadre of clinician scientists is a necessary foundation. (Bullmore et al., 2009; Lehner and Insel, 2010) The NIH Neuroscience Blueprint has announced grants targeting neuroscience education prior to entering university, emphasizing neuroscience training at an early stage. Training programs in psychiatry should focus on integrating basic and clinical neuroscience, translational medicine and novel methodologies. These principles are incorporated into the One Mind project (www.1Mind4Research.org). It is also vital that the institutional knowledge built up in industry and academic drug discovery teams is not lost. Knowledge manifest in key individuals in industry may need to be protected by encouraging re-employment of key individuals in academic posts.

The public perception of mental health and pharmaceutical research is crucial to the support of this vision. The role of government should be to work together with interest groups, including patient advocacy groups, to facilitate rapid development in translation into practice of novel, safe and effective treatments. As with other areas of medicine, these treatments may be optimized by targeting at specific subgroups, identified through biomarkers and endophenotypes. Private-public partnerships working closely with patient groups and government could greatly improve public access for specific groups of patients to effective treatments for specific symptoms. Financial incentives for innovation are required along with addressing legislation to protect brand, know-how and patents.

The Royal Society Seminar addressing these challenges in drug development resulted in a series of initiatives: The Institute of Medicine (USA) will facilitate meetings to develop the infrastructure to place drugs in a publically accessible space – a ‘medicine cabinet’ – and investigate legal issues surrounding intellectual property rights and insurance. Here we outline a series of novel mechanisms with preclinical validity in psychiatric disorders with high potential for further development by industry (Table 1). The table provides some examples which highlight the bi-directional communication necessary for a successful academia-industry partnership and will enhance knowledge of and access to the compounds that may be eligible for ‘repurposing’ and ‘rescue’, although they may not have been successful for their initial clinical indication.

Neuroscience is well poised for high impact discoveries, which have real possibilities to improve functional outcome and wellbeing of patients with mental health problems. It should be possible to have a vision of a holistic treatment for these debilitating psychiatric disorders that will significantly benefit patient outcome, as well as the economy. The issue of drug discovery underscores infrastructure issues in mental health research. Whilst some of these issues are being aggressively addressed and were recently highlighted (Insel and Sahakian, 2012), much more needs to be done. The aim of this paper is to provide a focus as to how to progress discussion toward achieving this obtainable vision over the next two decades.

## Figures and Tables

**Table 1:** Novel mechanisms with preliminary clinical validation: examples for a toolbox for mechanistic studies.

Class	Mechanism	Indication	References
Glutamate based therapies	NMDA antagonists (ketamine like)	Major depressive disorder	(Zarate et al., 2010)
	NMDA NR2b blockers	Major depressive disorder	(Preskorn et al., 2008)
	Metabotropic glutamate agonists (mGluR2-3)	Schizophrenia	(Patil et al., 2007)
	Glycine transport (GlyT1) blockers	Schizophrenia—negative symptoms	(Pinard et al., 2010)

Modulation of other Neurotransmitters	Serotonin-6 (5HT6) blockers	Cognitive symptoms in depression and schizophrenia	(Maher-Edwards et al., 2010)
	Alpha7-nicotinic agonists	Cognitive symptoms in AD and schizophrenia	(Tregellas et al., 2011)
	Histamine-3	Cognition and ADHD	(Schwartz, 2011)
	Muscarinic (M1) agonists	Cognitive symptoms in AD and schizophrenia	(Bodick et al., 1997)
	GABA A alpha 2,3 selective agonists	Anxiety, schizophrenia	(Lewis et al., 2008)
	Triple reuptake inhibitor (5HT, NE, DA	Major depressive disorder	(Tran et al., 2011)
